# Assessing the predictive value of neutrophil percentage to albumin ratio for ICU admission in ischemic stroke patients

**DOI:** 10.3389/fneur.2024.1322971

**Published:** 2024-02-01

**Authors:** Mohammed Zawiah, Amer Hayat Khan, Rana Abu Farha, Abubakar Usman, Fahmi Y. Al-Ashwal, Mohammed Ahmed Al-Kaif

**Affiliations:** ^1^Department of Clinical Pharmacy, College of Pharmacy, Northern Border University, Rafha, Saudi Arabia; ^2^Discipline of Clinical Pharmacy, School of Pharmaceutical Sciences Universiti Sains Malaysia, Penang, Malaysia; ^3^Department of Clinical Pharmacy and Therapeutics, Faculty of Pharmacy, Applied Science Private University, Amman, Jordan; ^4^Department of Clinical Pharmacy and Practice, College of Pharmacy, QU Health, Qatar University, Doha, Qatar; ^5^Department of Clinical Pharmacy, College of Pharmacy, Al-Ayen Iraqi University, Thi-Qar, Iraq; ^6^Department of Cardiology, QingPu Branch of Zhongshan Hospital Affiliated to Fudan University, Shanghai, China

**Keywords:** acute ischemic stroke, neutrophil percentage to albumin ratio, intensive care unit, neutrophil-to-lymphocyte ratio, predictive value

## Abstract

**Background:**

Acute ischemic stroke (AIS) remains a substantial global health challenge, contributing to increased morbidity, disability, and mortality. This study aimed at investigating the predictive value of the neutrophil percentage to albumin ratio (NPAR) in determining intensive care unit (ICU) admission among AIS patients.

**Methods:**

A retrospective observational study was conducted, involving AIS cases admitted to a tertiary hospital in Jordan between 2015 and 2020. Lab data were collected upon admission, and the primary outcome was ICU admission during hospitalization. Descriptive and inferential analyses were performed using SPSS version 29.

**Results:**

In this study involving 364 AIS patients, a subset of 77 (21.2%) required admission to the ICU during their hospital stay, most frequently within the first week of admission. Univariable analysis revealed significantly higher NPAR levels in ICU-admitted ischemic stroke patients compared to those who were not admitted (23.3 vs. 15.7, *p* < 0.001), and multivariable regression models confirmed that higher NPAR (≥19.107) independently predicted ICU admission in ischemic stroke patients (adjusted odds ratio [aOR] = 4.85, 95% CI: 1.83–12.83). Additionally, lower GCS scores and higher neutrophil-to-lymphocyte ratio (NLR) were also associated with increased likelihood of ICU admission. In terms of predictive performance, NPAR showed the highest accuracy with an AUC of 0.885, sensitivity of 0.805, and specificity of 0.854, using a cutoff value of 19.107. NPAR exhibits an AUC of 0.058, significantly outperforming NLR (*Z* = 2.782, *p* = 0.005).

**Conclusion:**

NPAR emerged as a robust independent predictor of ICU admission in ischemic stroke patients, surpassing the predictive performance of the NLR.

## Introduction

Acute ischemic stroke (AIS) remains a substantial global health challenge, contributing to increased morbidity (1, 2), disability (3), and mortality (4, 5). While many cases of AIS can be efficiently treated in regular hospital environments or specialized stroke units, it is worth noting that approximately quarter of AIS patients require admission to an intensive care unit (ICU) (3, 6). These ICU admissions are crucial due to the severity of the condition. Importantly, the reported mortality rates for stroke patients receiving treatment in the ICU exhibit a wide range, varying from 14% to as high as 70% (1, 3–5), underscoring the need for comprehensive and targeted care strategies for this patient population.

Predicting ICU admission in acute ischemic stroke (AIS) patients is crucial for optimizing care and resource allocation. While the National Institutes of Health Stroke Scale (NIHSS) is commonly used for severity assessment, it has limitations. Solely relying on NIHSS for ICU admission may offer no cost or outcome benefits for mild to moderate stroke cases (7). Furthermore, its reliance on detailed neurological assessments, often unavailable in-patient records, could limit its applicability (8) and may not capture specific stroke symptoms’ impact (8). Modifications, especially for posterior circulation stroke, where NIHSS is less effective, may be necessary (9). Additionally, low NIHSS scores in acute ischemic stroke with symptomatic arterial occlusion may not signify mild stroke during the acute phase (10).

Inflammation is part of the initial brain damage after a stroke and contributes to tissue repair in the stroke’s later phases (11). Measuring peripheral leukocytes, particularly neutrophils, provided an affordable and accessible means to evaluate the potential presence of inflammation (12). In the initial phases following a stroke, neutrophils migrate to the brain area affected by the infarction and undergo activation. This activation prompts the release of various substances like reactive oxygen and nitrogen species, chemokines, and neutrophil extracellular traps. These substances have the potential to induce additional inflammation, attracting and activating other immune cells in the process (13). Albumin, a protein of moderate size, has a molecular weight ranging from 66 to 69 kilodaltons, constituting over half of the total composition of proteins within the serum (14). Albumin serves various roles such as osmoregulation, antioxidative action, and anti-inflammatory properties (15, 16).

Recent research has emphasized the utility of objective, cost-effective, and readily available laboratory-based tests like the Neutrophil-to-Lymphocyte Ratio (NLR), recognizing its potential as valuable tool in predicting severity and prognostic outcomes for AIS patients (17, 18). Furthermore, the Neutrophil Percentage-to-Albumin Ratio (NPAR) has recently gained attention for its significant role in predicting post stroke complications and mortality of stroke patients (19, 20). However, despite its recognized importance in stroke prognosis, there has been a notable gap in research regarding its potential utility in predicting the admission of ischemic stroke patients to the ICU. This study aimed to address this gap by investigating the predictive value of the NPAR specifically in determining ICU admission among ischemic stroke patients.

## Methods

### The study subjects

This retrospective cohort study focused on patients with acute ischemic stroke admitted to Jordan University Hospital in Amman, Jordan, spanning from beginning of 2015, to the end of 2020. To be eligible for inclusion, patients needed to be adults aged 18 years and older, possess a complete medical record, and have a confirmed physician diagnosis of acute ischemic stroke based on neurologic examination and radiologic confirmation that aligned with the criteria specified by the World Health Organization (21, 22). Exclusions from the study were made for patients with advanced cancer or haematological diseases, individuals with a history of corticosteroid or immunosuppressant use within the 3 months prior to the stroke, patients with chronic liver diseases, and those who were transferred to another hospital.

In adherence to ethical principles, this study was conducted in accordance with the Helsinki Declaration. The research protocol received approval from the JUH Institutional Review Board under reference number 10-2021-4345. Patient consent requirements were waived because of the retrospective nature of the study, as authorized by the ethical committee approval.

### Data screening and collection

During the study period, a thorough review of electronic medical records was conducted to identify eligible participants. From the eligible records, demographic information such as age, sex, and the duration of hospitalization were firstly reported. Relevant pre-existing risk factors such as current smoking and comorbidities like ischemic heart disease, chronic heart failure, hypertension, diabetes, previous stroke, and atrial fibrillation were meticulously documented. Vital signs and clinical data, including systolic and diastolic blood pressure, respiratory rate, heart rate, oxygen saturation, body temperature, and the Glasgow Coma Scale (GCS), were also recorded. Regarding Laboratory Parameters, within the initial 24 h of admission or emergency visit, a comprehensive set of laboratory data was collected. This included complete blood counts, encompassing neutrophils, lymphocytes, monocytes, eosinophils, basophils, hematocrit (Hct), hemoglobin (Hb), red blood cell (RBC) counts, white blood cell (WBC) counts, and platelet counts. Additionally, levels of albumin, serum creatinine, and blood urea nitrogen were measured. Using these laboratory results, several ratios were computed such as the neutrophil-to-lymphocyte ratio (NLR), and the neutrophil percentage to albumin ratio (NPAR). The NLR is calculated by dividing the absolute neutrophil count by the absolute lymphocyte count in a given blood sample, and the NPAR is derived by dividing total neutrophil count divided by total serum albumin measured in grams per decilitre. The included patients were classified based on the occurrence of the primary outcome of interest, which was admission to the ICU. This was determined by any documented admission to the ICU during the hospitalization.

### Statistical analysis

The statistical analysis was conducted using SPSS version 29. Categorical variables were presented as frequencies and percentages, while continuous variables were expressed as medians with interquartile ranges due to their non-normal distribution. Univariable analyses were conducted with two different groupings: one based on ICU admission and the other based on high and low NPAR levels. Categorical variable associations were investigated using the Pearson Chi-square test, and differences in continuous variables between the two groups were assessed using the Mann–Whitney U test. Variables that showed statistical significance in the univariable analyses (*p* < 0.05) were subsequently included in a multivariable logistic regression model, using a backward stepwise method, to identify independent predictors of ICU admission in acute ischemic stroke patients. Three distinct multivariable models were developed. The first two utilized logistic regression: one treated NPAR as a continuous variable, while the other categorized NPAR based on a cutoff point of 19.07, determined by maximizing the Youden index. The third model employed categorized NPAR in a time-to-event analysis using Cox regression. To assess the model’s performance and NPAR’s predictive ability, discrimination measures were calculated, including the C-statistics (AUC ROC) with a 95% confidence interval (CI). Additionally, NPAR’s predictive power was compared with other ratios, such as NLR, using the DeLong test to evaluate AUC differences. Optimal cutoff values for these ratios were determined by maximizing Youden’s index (sensitivity + specificity − 1). Model calibration was assessed using the Hosmer-Lemeshow test, where a non-significant *p*-value (≥0.05) and a smaller chi-square value indicated good model calibration.

## Results

### Sociodemographic and clinical characteristics of the patients

**Figure 1:**
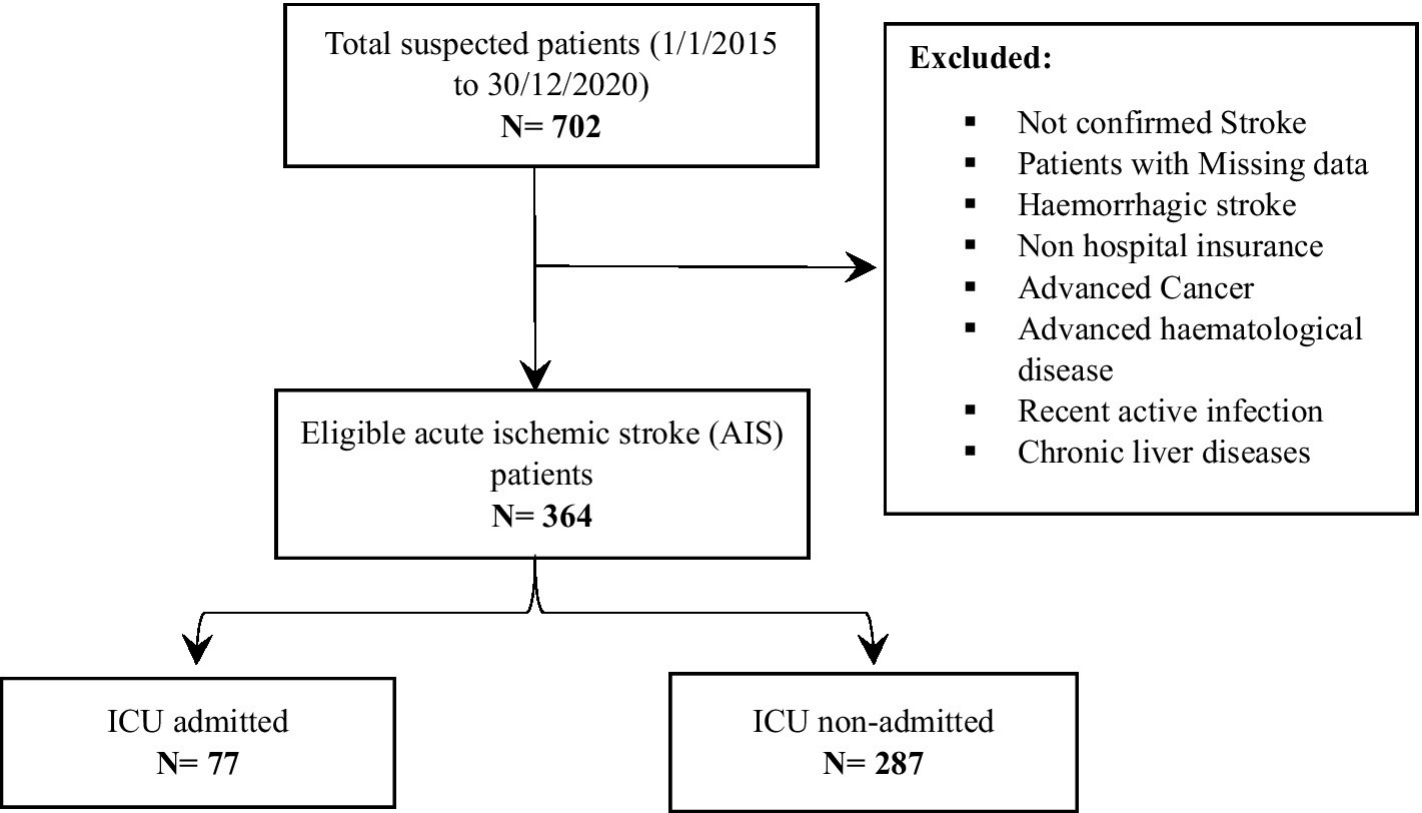
The study flowchart.

Throughout the study period, 364 ischemic stroke patients were included (Figure 1). The baseline sociodemographic and clinical characteristics of the recruited patients illustrated in the Table 1. Overall, the median age was 71 years, with an interquartile range (IQR) spanning from 61 to 77 years. Of the patients, 41.8% were male, and 21.4% of them being smokers. Out of the total patients, 77 (21.2%) required admission to the ICU during their hospital stay, with a median ICU admission time of 3 days. It is noteworthy that the vast majority of ICU admissions (90.7%) occurred within the first week after admission.

**Table 1:** Sociodemographic and clinical characteristics of the patients.

Factor	Total (364)	Non-ICU (287)	ICU (77)	*p* value
Age (years), median [IQR]	71 (61, 77)	70 (60, 76)	75 (63, 80)	**0.001**
Gender (Male) [*n* (%)]	152 (41.8)	116 (40.4)	36 (46.8)	0.317
BMI (Kg/m^2^), median [IQR]	27.7 (25.7, 31.2)	27.7 (25.7, 30.8)	29.0 (25.8, 31.6)	0.188
Smoking (yes) [*n* (%)]	77 (21.4)	65 (22.7)	12 (16.4)	0.243
**Co-morbidities**				
HTN [*n* (%)]	292 (80.2)	234 (81.5)	58 (75.3)	0.225
DM [*n* (%)]	220 (60.4)	174 (60.6)	46 (59.7)	0.888
IHD [*n* (%)]	99 (27.2)	758 (26.1)	24 (31.2)	0.378
Prior stroke/TIA [*n* (%)]	74 (20.4)	55 (19.2)	19 (25.0)	0.261
AFIB [*n* (%)]	41 (11.3)	24 (8.4)	17 (22.1)	**<0.001**
CHF [*n* (%)]	31 (8.5)	19 (6.6)	12 (15.6)	**0.012**
**Vital signs**				
Temperature (°C)	36.7 (36.5, 37)	36.7 (36.5, 37)	36.7 (36.6, 37)	0.760
Systolic BP (mmHg), median [IQR]	145 (130, 164)	144 (130, 161)	147 (126, 172)	0.685
Diastolic BP (mmHg), median [IQR]	80 (72, 90)	81 (72, 90)	80 (71.75, 90)	0.526
Pulse rate (BPM), median [IQR]	80 (73, 88)	80 (74, 88)	80 (71, 89)	0.866
Respiratory rate, median [IQR]	20 (18, 20)	20 (18, 20)	20 (18, 22)	0.107
O2sat (%)	96 (95, 98)	96 (95, 98)	95 (93, 97)	**<0.001**
GCS score, median [IQR]	15 (15, 15)	15 (15, 15)	13 (10, 15)	**<0.001**
**Laboratory data**				
RBS, median [IQR]	144 (111, 222)	143 (108, 227)	152 (119, 207)	0.295
WBC (× 10^9^ /L), median [IQR]	8.4 (7.0, 10.0)	8.3 (6.8, 9.9)	8.78 (7.49, 11.43)	**0.005**
Neutrophil %, median [IQR]	67.0 (58.0, 75.4)	64.6 (56.8, 71.0)	78 (70.05, 84.9)	**<0.001**
Lymphocyte %, median [IQR]	23.4 (16.7, 31.0)	25.9 (20.3, 32.4)	12.5 (8.4, 20.6)	**<0.001**
Monocyte %, median [IQR]	5.5 (4.4, 7.0)	5.5 (4.5, 7)	5.3 (4, 7)	0.263
Eosinophil %, median [IQR]	1.9 (1, 2.9)	2 (1.1, 3)	1.1 (0.48, 2.1)	**<0.001**
Basophil %, median [IQR]	0.5 (0.3, 0.8)	0.6 (0.3, 0.8)	0.3 (0.2, 0.5)	**<0.001**
PLT (× 10^9^ /L), median [IQR]	248 (204, 294)	251 (208, 301)	227 (193.5, 272)	**0.008**
RBC (× 10^12^ /L), mean [SD]	4.6 (4.2, 5.0)	4.6 (4.2, 5.1)	4.4 (3.8, 4.9)	**0.001**
Hb (g/dL), median [IQR]	13.1 (11.9, 14.4)	13.4 (12.2, 14.6)	12.5 (11.0, 13.6)	**<0.001**
Hct %, median [IQR]	39.6 (36.4, 43.3)	40 (36.8, 44.0)	38.2 (34.0, 41.7)	**<0.001**
NLR, median [IQR]	2.8 (1.9, 4.7)	2.49 (1.7, 3.6)	6.4 (3.6, 10.0)	**<0.001**
NPAR, median [IQR]	16.3 (14.1, 19.9)	15.7 (13.8, 17.8)	23.3 (20.9, 27.5)	**<0.001**
Albumin (g/dL), median [IQR]	4.1 (3.7, 4.2)	4.2 (4, 4.2)	3.5 (2.9, 3.9)	**<0.001**
Scr(mg/dL), median [IQR]	0.82 (0.65, 1.1)	0.80 (0.64, 1.05)	0.95 (0.72, 1.34)	**0.003**
BUN, median [IQR]	17.7 (13.4, 23.4)	16.8 (13.1, 21.5)	22.9 (14.1, 31.8)	**<0.001**
**Other**				
Hospitalization, median [IQR]	4 (2, 7)	3 (2, 5)	11 (6, 22)	**<0.001**

AFIB, atrial fibrillation; BMI, body mass index; BP, blood pressure; BUN, blood urea nitrogen; CHF, congestive heart failure; DM, diabetes mellitus; GCS, Glasgow Coma Scale; Hb, hemoglobin; Hct, hematocrit; HTN, hypertension; ICU, intensive care unit; IHD, ischemic heart disease; IQR, interquartile range; NLR, neutrophil to lymphocyte ratio; NPAR, neutrophil percentage to albumin ratio; O2sat, oxygen saturation; PLT, platelet; RBC, red blood cells; SAP, stroke associated pneumonia; TIA, transient ischemic attack; WBC, white blood cells.

### Comparison of clinical characteristics between ICU and non-ICU patients

Significant differences between ICU-admitted and non-ICU-admitted ischemic stroke patients include age (ICU: 75 years, non-ICU: 70 years, *p* = 0.001), the prevalence of atrial fibrillation (ICU: 22.1%, non-ICU: 8.4%, *p* < 0.001), congestive heart failure (ICU: 15.6%, non-ICU: 6.6%, *p* = 0.012), lower oxygen saturation in ICU patients (ICU: 95%, non-ICU: 96%, *p* < 0.001), and lower GCS in ICU patients (median GCS score: ICU: 13, non-ICU: 15, *p* < 0.001). ICU patients had significantly higher levels of RBS, WBC count, Neutrophil percentage, and Basophil percentage but lower levels of Lymphocyte percentage, Hemoglobin, Hematocrit, and Albumin. Moreover, Scr levels, BUN levels, and hospitalization duration were notably higher in ICU patients.

### Comparison of clinical characteristics between patients with low and high NPAR levels

Patients with high NPAR levels (≥19.107), compared to those with low NPAR, were older (median age 75 vs. 68 years), had lower GCS scores (median GCS 14 vs. 15), and a higher prevalence of comorbidities such as atrial fibrillation (19.2% vs. 8.0%) and congestive heart failure (14.4% vs. 6.1%). High NPAR patients also displayed elevated neutrophil percentages (median 74.8% vs. 65.8%) and reduced lymphocyte percentages (median 12.4% vs. 27.5%). They had lower albumin levels (median 3.5 vs. 4.2 g/dL) and higher serum creatinine levels (median 0.97 vs. 0.78 mg/dL). High NPAR patients had longer hospitalizations (median 9 vs. 3 days), signifying a greater need for extended medical care (Table 2).

**Table 2:** Baseline characteristics of the patients in the high (≥19.107) and low NPAR (<19.107).

Factor	Low NPAR (261)	High NPAR (104)	*p* value
Age (years), median [IQR]	68 (59, 75)	75 (69, 80)	**<0.001**
Gender (Male) [*n* (%)]	108 (41.4)	44 (42.3)	0.871
BMI (Kg/m^2^), median [IQR]	27.7 (25.9, 31.2)	27.6 (24.6, 31.2)	0.410
Smoking (yes) [*n* (%)]	58 (22.3)	19 (19.0)	0.493
**Co-morbidities**			
HTN [*n* (%)]	209 (80.1)	83 (79.8)	0.954
DM [*n* (%)]	156 (59.8)	64 (61.5)	0.755
IHD [*n* (%)]	71 (27.2)	28 (26.9)	0.957
Prior stroke/TIA [*n* (%)]	49 (18.8)	26 (25.2)	0.169
AFIB [*n* (%)]	21 (8.0)	20 (19.2)	**0.002**
CHF [*n* (%)]	16 (6.1)	15 (14.4)	**0.010**
**Vital signs**			
Temperature (°C)	36.7 (36.5, 37.0)	36.7 (36.6, 36.9)	0.634
Systolic BP (mmHg), median [IQR]	145 (130, 162)	146 (130, 167)	0.691
Diastolic BP (mmHg), median [IQR]	81 (73, 90)	80 (70, 90)	0.160
Pulse rate (BPM), median [IQR]	80 (73, 88)	80 (73, 89)	0.527
Respiratory rate, median [IQR]	20 (18, 20)	20 (18, 20)	0.313
O2sat (%)	96 (95, 98)	95 (94, 97)	**0.002**
GCS score, median [IQR]	15 (15, 15)	14 (11, 15)	**<0.001**
**Laboratory data**			
RBS, median [IQR]	136 (103, 218)	163 (121, 238)	0.015
WBC (× 10^9^ /L), median [IQR]	8.1 (6.6, 9.7)	9.3 (7.7, 11.5)	**0.005**
Neutrophil %, median [IQR]	65.8 (56.3, 68.7)	74.8 (80.4, 84.2)	**<0.001**
Lymphocyte %, median [IQR]	27.5 (22.3, 33.0)	12.4 (9.0, 17.9)	<0.001
Monocyte %, median [IQR]	5.6 (4.7, 7.2)	5.0 (4.0, 6.1)	<0.001
Eosinophil %, median [IQR]	2.2 (1.3, 3.0)	1.0 (0.4, 1.8)	<0.001
Basophil %, median [IQR]	0.6 (0.4, 0.9)	0.3 (0.2, 0.5)	<0.001
PLT (× 109 /L), median [IQR]	250 (209, 301)	232 (187, 281)	0.036
RBC (× 1,012 /L), mean [SD]	4.7 (4.3, 5.2)	4.4 (3.9, 4.8)	<0.001
Hb (g/dL), median [IQR]	13.4 (12.3, 14.6)	12.6 (11.2, 13.6)	**<0.001**
Hct %, median [IQR]	40.3 (37.3, 44.1)	37.3 (34.2, 40.2)	**<0.001**
NLR, median [IQR]	2.7 (1.7, 3.0)	6.5 (4.5, 9.4)	**<0.001**
Albumin (g/dL), median [IQR]	4.2 (4, 4.3)	3.5 (3, 3.8)	**<0.001**
Scr (mg/dL), median [IQR]	0.78 (0.63, 1.0)	0.97 (74, 1.3)	**<0.001**
BUN, median [IQR]	16.8 (13.0, 21.0)	21.7 (14.0, 28.3)	**<0.001**
**Others**			
Hospitalization, median [IQR]	3 (2, 5)	9 (4, 18)	**<0.001**
ICU [*n* (%)]	15 (5.8)	62 (59.6)	**<0.001**

AFIB, atrial fibrillation; BMI, body mass index; BP, blood pressure; BUN, blood urea nitrogen; CHF, congestive heart failure; DM, diabetes mellitus; GCS, Glasgow Coma Scale; Hb, hemoglobin; Hct, hematocrit; HTN, hypertension; ICU, intensive care unit; IHD, ischemic heart disease; IQR, interquartile range; NLR, neutrophil to lymphocyte ratio; NPAR, neutrophil percentage to albumin ratio; O2sat, oxygen saturation; PLT, platelet; RBC, red blood cells; SAP, stroke associated pneumonia; TIA, transient ischemic attack; WBC, white blood cells.

### Association between NPAR and ICU admission of ischemic stroke patients

**Figure 2:**
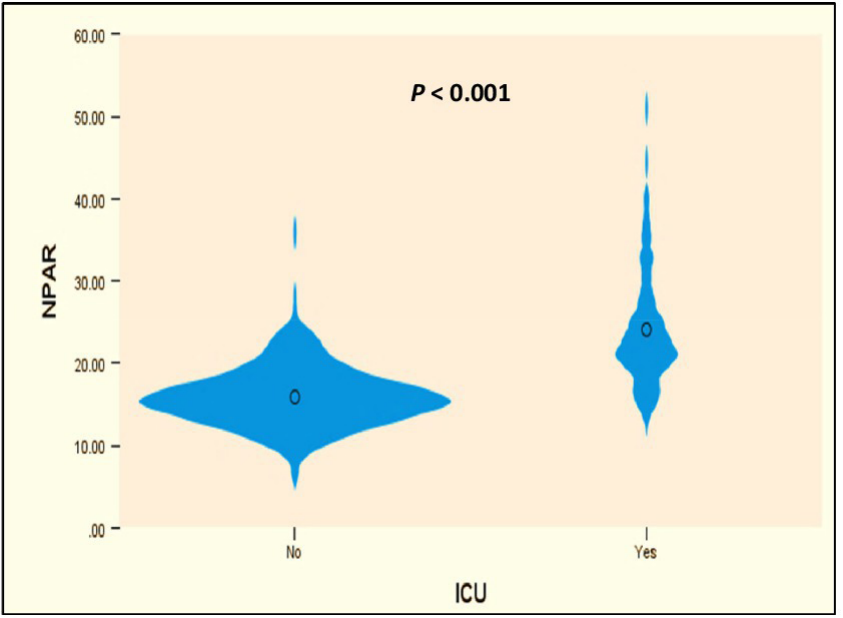
Violin plot of NPAR levels and distributions in ICU and non-ICU patients.

**Figure 3:**
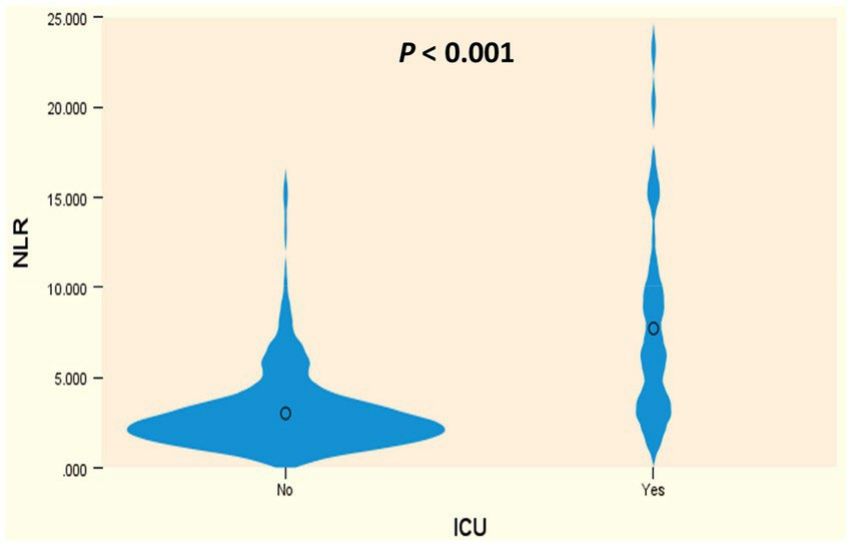
Violin plot of NLR levels and distributions in ICU and non-ICU patients.

Initially, univariable logistic analysis revealed significantly higher NPAR levels in ICU-admitted ischemic stroke patients compared to those who were not admitted (23.3 vs. 15.7, *p* < 0.001) (Figure 2). Subsequently, after accounting for potential confounding factors (identified with *p* values <0.05 in the univariable analysis), a multivariable regression model (Model 1, Table 3) demonstrated that NPAR remained independently associated with an increased likelihood of ICU admission (B = 0.217, *p* < 0.001). Moreover, in an additional multivariable regression model (Model 2, Table 3), which incorporated NPAR categorized based on a specific cutoff point (≥19.107), it was confirmed that higher NPAR independently predicted ICU admission in ischemic stroke patients (adjusted odds ratio [aOR] = 4.85, 95% CI: 1.83–12.83) while adjusting for the same confounding variables. Cox regression analysis designated as “model 3,” found that the higher NPAR level (≥19.107) increase the likelihood of admission of ischemic stroke patients to the ICU (adjusted hazard ratio [HR] = 5.06, 95% CI, 2.45–10.44: *p* value, < 0.001). In addition to the NPAR, higher NLR (B = 0.320, *p* < 0.001) found to be increased likelihood of ICU admission in both univariable (Figure 3) and multivariable analyses. Furthermore, lower GCS scores (B = −0.525, *p* < 0.001) was also found to be independently predict the admission to ICU. The Hosmer and Lemeshow test indicated good calibration for both models (Model 1: χ^2^ = 10.297, *p* = 0.245; Model 2: χ^2^ = 6.649, *p* = 0.575).

**Table 3:** Multivariable regression analyses of independent predictors for ICU admission in ischemic stroke patients.

	Model 1			Model 2			Model 3		
Factor	B	OR (95% CI)	*p*	B	OR (95% CI)	*p*	B	HR (95% CI)	*p*
GCS	−0.585	0.56 (0.43–0.72)	**<0.001**	−0.525	0.59 (0.46–0.77)	**<0.001**	−0.175	0.84 (0.77–0.92)	**<0.001**
Eosinophil	0.190	1.21 (0.98–1.50)	0.058	0.193	1.21 (0.98–1.50)	0.077	–	–	–
CHF	0.970	2.64 (0.87–8.03)	0.088	–	–	–	–	–	–
O2 saturation	–	–	–	−0.113	0.89 (0.79–1.01)	0.065	−0.066	0.94 (0.98–1.00)	0.051
NLR	0.272	1.31 (1.13–1.53)	**<0.001**	0.320	1.37 (1.18–1.61)	**<0.001**	0.079	1.08 (1.03–1.14)	**0.002**
Monocyte	0.180	1.20 (0.98–1.47)	0.083	0.173	1.19 (0.97–1.50)	0.092	–	–	–
NPAR	0.217	1.24 (1.11–1.39)	**<0.001**	–	–	–	–	–	–
Higher NPAR (≥19.107)	–	–	–	1.579	4.85 (1.83–12.83)	**0.001**	1.621	5.06 (2.45–10.44)	**0.001**

Both models included variables that had a *p*-value of less than 0.05 in the univariable analysis including Age, GCS, Afib, CHF, O2 Saturation, WBC, Lymphocyte, Monocyte, Eosinophil, Basophil, RBC, HCT, NLR, BUN, HB, and PLT. Model 1 included NPAR as a scale and utilized logistic regression. Model 2 incorporated NPAR categorized based on a cutoff point (19.107) and employed logistic regression. Model 3 involved NPAR categorized based on a cutoff point (19.107) and utilized Cox regression. BUN, blood urea nitrogen; CHF, chronic heart failure; CI, confidence interval; GCS, Glasgow Coma Scale; HR, hazard ratio; NLR, neutrophil to lymphocyte ratio; NPAR, neutrophil percentage to albumin ratio; OR, odds ratio.

### The predictive performance of NPAR

**Figure 4:**
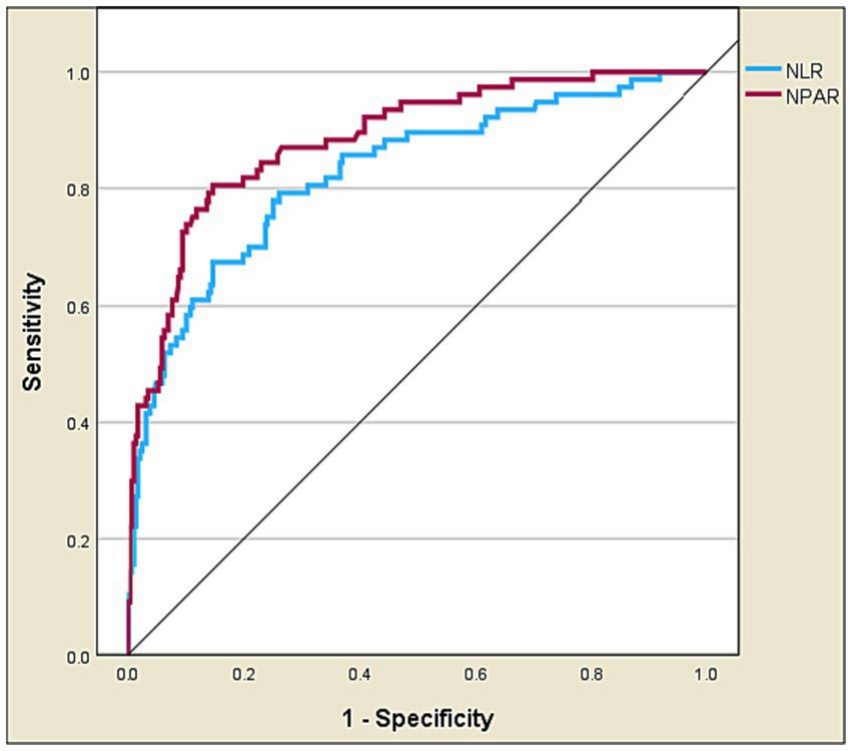
Receiver operating characteristic curve analysis of NPAR vs. NLR in predicting ICU admission of ischemic stroke patients.

**Figure 5:**
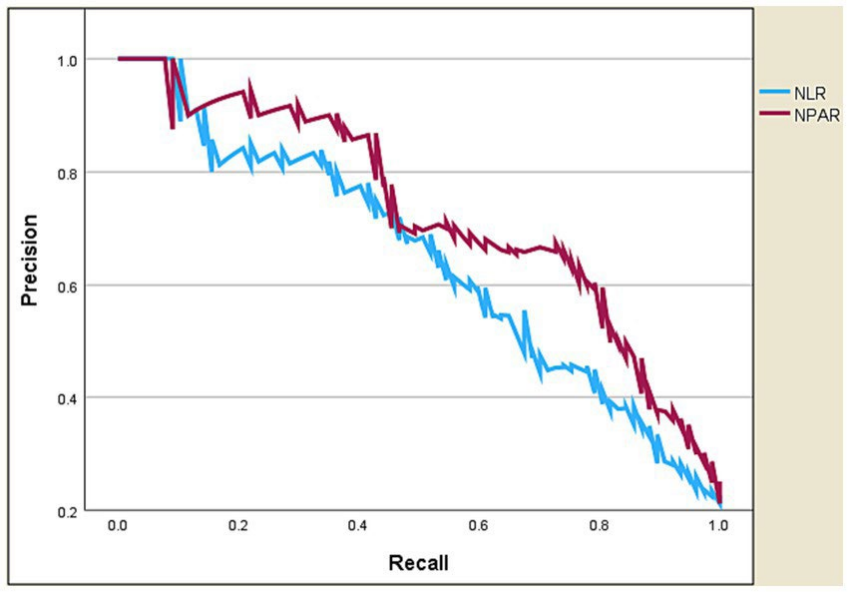
Precision-recall curve of NPAR vs. NLR in predicting ICU admission of ischemic stroke patients.

Table 4 and Figures 4, 5 present the predictive performance of various blood-based ratios in ICU admission of ischemic stroke patients. NPAR exhibited the highest performance, with an AUC of 0.885, sensitivity of 0.805, and specificity of 0.854, using a cut-off value of 19.107. NLR also performed well, with an AUC of 0.827, sensitivity of 0.675, and specificity of 0.854 at a cut-off value of 4.524. The DeLong test results (Table 5) demonstrate significant differences in the predictive values between these ratios. NPAR exhibits an AUC of 0.058, significantly outperforming NLR (*Z* = 2.782, *p* = 0.005).

**Table 4:** Predictive performance and optimal cut-off point for NPAR and NLR.

Predictor	AUC	95% CI	*p* value	Optimal cut-off value	Sensitivity	Specificity	YI
NPAR	0.885	0.842–0.927	<0.001	19.107	0.805	0.854	0.659
NLR	0.827	0.771–0. 882	<0.001	4.524	0.675	0.854	0.529
Albumin	0.844	0.791–0.896	<0.001	3.850	0.740	0.805	0.545
Neutrophil	0.813	0.757–0.869	<0.001	73.45	0.688	0.822	0.511

AUC, area under the curve; CI, confidence interval; NPAR, neutrophil percentage to albumin ratio; NLR, neutrophil to lymphocyte ratio; ROC, receiver operating characteristic curve; SE, standard error; YI, Youden’s index.

**Table 5:** Area difference under the ROC curves for NPAR vs. NLR.

Predictors pairs	*Z*	*p* value	AUC difference	SE difference	95% CI
NPAR – NLR	2.782	0.005	0.058	0.222	(0.017–0.099)

AUC, area under the curve; CI, confidence interval; NLR, neutrophil to lymphocyte ratio; NPAR, neutrophil percentage to albumin ratio; ROC, receiver operating characteristic curve; SE, standard error.

## Discussion

This is the first study to assess the predictive value of NPAR in determining the need for ICU admission in AIS patients. Our findings revealed important insights. Firstly, NPAR was established as a robust and independent predictor of ICU admission. Secondly, when comparing NPAR and NLR, it became evident that NPAR exhibited significantly higher predictive performance for ICU admission than NLR.

NPAR has been investigated in prior studies and has consistently demonstrated clinical relevance. This study mirrors recent investigations that have shown NPAR to be associated with early neurological deterioration and reflective of the severity of acute ischemic stroke (23, 24). These findings indicate that NPAR could serve as an indicator of the rapidity of neurological decline in stroke patients, which, in turn, could contribute to the need for ICU care. Furthermore, our observation of NPAR’s significance aligns with research linking it to infection incidence, particularly stroke-associated pneumonia which commonly occur in the first week of stroke onset (25, 26). This association suggests that elevated NPAR levels may reflect an underlying inflammatory response triggered by infections, making it a valuable marker for identifying patients at a higher risk of complications, such as those necessitating ICU admission. In essence, the current study provides further validation of the clinical utility of NPAR by demonstrating its superiority, even when compared to the previously studied NLR, in predicting ICU admission. It underscores the comprehensive nature of NPAR as a predictive marker, encompassing crucial aspects of stroke prognosis, and thereby substantiates its value in risk stratification and clinical decision-making for ischemic stroke patients.

The study findings may be underpinned by several mechanistic considerations. Previous studies have shed light on the potential mechanisms that underscore the clinical associations we observed. Firstly, albumin, a major component of the NPAR calculation, is known to exert neuroprotective functions through various pathways (27, 28). It possesses anti-inflammatory properties, which can mitigate the inflammatory response within the brain following ischemic events. Additionally, albumin’s antioxidant characteristics help counteract oxidative stress, a pivotal factor in stroke pathophysiology and severity (14, 29–31). Furthermore, albumin has been shown to inhibit endothelial apoptosis and regulate microvascular permeability, contributing to the maintenance of cerebrovascular integrity (27, 32). Neutrophils, in response to cerebral ischemia, are rapidly recruited to the ischemic site, while microglia become activated (33). Neutrophils release a multitude of substances, including reactive oxygen species, inflammatory mediators, chemokines, cytokines, adhesion molecules, and proteases. This collective release of factors plays a pivotal role in the disruption of the blood–brain barrier (BBB), ultimately exacerbating ischemic damage and contributing to brain edema (34, 35). Furthermore, it is noteworthy that neutrophils are recognized as a significant source of matrix metalloproteinase-9, an enzyme that has been closely associated with BBB breakdown and the occurrence of hemorrhagic transformation (36, 37). This intricate interplay of neutrophil-driven inflammation and its impact on BBB integrity underscores the potential mechanisms that contribute to adverse outcomes in cases of cerebral ischemia.

We could not adjust for the NIHSS in this study due to its unavailability. This limitation has been noted in previous research as well (38, 39). To address this, we used the GCS as alternative way to estimate stroke severity. Moreover, it was carried out at a single center, which may introduce selection bias due to the relatively small sample size. Future research should consider conducting multicentre, prospectively designed studies to address these limitations. Despite these limitations, our study is the first to investigate a new biomarker called NPAR, which could be valuable for predicting ICU admission in ischemic stroke patients.

## Conclusion

The findings of our study demonstrated that NPAR is a robust independent predictor of ICU admission in ischemic stroke patients, surpassing the predictive performance of NLR. NPAR’s enhanced predictive power stems from incorporating albumin levels, providing a more comprehensive assessment of inflammatory status and overall health and nutrition. This nuanced approach could improve accuracy in predicting ICU admission, indicating the potential clinical relevance of NPAR over traditional NLR. However, further validation in larger cohorts is necessary to confirm its broader applicability.

## Data availability statement

The raw data supporting the conclusions of this article will be made available by the authors, without undue reservation.

## Ethics statement

The studies involving humans were approved by JUH Institutional Review Board under reference number 10-2021-4345. The studies were conducted in accordance with the local legislation and institutional requirements. Written informed consent for participation was not required from the participants or the participants' legal guardians/next of kin in accordance with the national legislation and institutional requirements.

## Author contributions

MZ: Conceptualization, Data curation, Formal analysis, Funding acquisition, Investigation, Methodology, Software, Writing – original draft. AK: Investigation, Methodology, Project administration, Validation, Visualization, Writing – review & editing. RF: Data curation, Methodology, Project administration, Software, Validation, Visualization, Writing – review & editing. AU: Data curation, Project administration, Resources, Validation, Writing – review & editing. FA-A: Conceptualization, Data curation, Investigation, Software, Visualization, Writing – review & editing. MA-K: Project administration, Supervision, Validation, Writing – review & editing.
